# The inhibition of Platycodin D on *Mycoplasma pneumoniae* proliferation and its effect on promoting cell growth after anti-*Mycoplasma pneumoniae* treatment

**DOI:** 10.3389/fcimb.2014.00192

**Published:** 2015-01-13

**Authors:** Yanli Meng, Yang Yang, Weihong Lu, Yingyan Wang, Feng Qian, Xin Wang, Zhihua Zhang, Weiming Wang

**Affiliations:** ^1^Heilongjiang Academy of Chinese Medicine SciencesHarbin, China; ^2^School of Food Science and Engineering, Harbin Institute of TechnologyHarbin, China

**Keywords:** *M. pneumoniae*, Platycodin D, A549 cells, mouse models, cell growth

## Abstract

Platycodin D, extract from the root of Platycodon grandiflorum, is one of the most important monomers of the Qinbaiqingfei pellets (Qinbai) that has already been approved as the first Traditional Chinese Medicine for clinic use as an anti-*M. pneumoniae* agent. Qinbai constituents Scutellaria baicalensis and Platycodon grandiflorum were used to treat thousands of patients clinically in China each year. In this study, a *M. pneumoniae*–infected mouse strain, BALB/c, and a human-derived epithelial cell line, A549 type II pneumocytes, were used as experimental model. Anti-*M. pneumoniae* effect of Platycodin D was measured by the Real-time quantitative PCR, while the cell pathological change with hematoxylin and eosin and the growth recovery effects were determined with 3-(4,5-dimethylthiazol-2-yl)-2,5-diphenyltetrazolium bromide and Trypan Blue dye in the experimental model after *M. pneumoniae* infection. Our research results showed that Platycodin D could significantly inhibit *M. pneumoniae* and promote cell growth after anti- *M. pneumoniae* treatment in the infected cells or mice.

## Introduction

*Mycoplasma pneumoniae (M. pneumoniae)* is the common pathogen for a variety of respiratory infections among school-age children. It is also a leading cause of all community-acquired pneumonia and other acute or chronic airway diseases (Waites and Talkington, 2004).

Because of *M. pneumoniae's* lack of cell wall, most antibiotics, including penicillin, are not effective in treating *M. pneumoniae* infections. Macrolides are still the most efficient drugs in treating *M. pneumoniae* infections due to their strong antimicrobial and anti-inflammatory properties. The mechanism of macrolides' antimicrobial action is the inhibition of bacterial protein synthesis after Macrolides are reversibly bound to the P site of the 50S subunit of the bacterial ribosome leading to the inhibition of polypeptide chain elongation and ribosomal translocation (Wikipedia, 2011).

Qinbai, a drug extracted from natural sources including Scutellaria baicalensis and Platycodon grandiflorum, is the first Chinese Traditional Medicine clinically used in treating *M. pneumoniae* infections in China. Qinbai phase II and III trials with 653 patients enrolled for 6 years were carried out by eight GCP labs. The trial results demonstrated that Qinbai was highly effective in treating *M. pneumoniae* without developing any adverse side effect or drug resistance, which is unpublished data. Early experiments also confirmed that Qinbai had high anti-*M. pneumoniae* pharmacodynamics *in vitro* with a MIC of 100 μg/ml. Qinbai can improve EGF expression in epithelial cells and prevent the damage to lung organizational structures caused by *M. pneumoniae*, leading to epithelial restoration and cilia regeneration (Meng et al., 2012). Further acute toxicity and chronic toxicity experiments showed that Qinbai was safe. Therefore, Qinbai became an alternative option besides Macrolides in treating *M. pneumoniae* infection.

Qinbai main constituents, Scutellaria baicalensis and Platycodon grandiflorum, have been used to treat thousands of patients in China. Platycodon grandiflorum was widely used to treat inflammatory diseases such as bronchitis, tonsillitis, laryngitis, and suppurative dermatitis. Platycodin D is extracted from the root of Platycodon grandiflorum and is one of monomers of Qinbaiqingfei pellets, while Platycodon grandiflorum has been widely used as Traditional Chinese Medicine for thousands years in treating various diseases. Recent study demonstrated that platycodin D enhanced anti-cancer activities of doxorubicin in both MCF-7 and MDA-MB-231 cell lines (Tang et al., 2014), induced cell apoptosis, and inhibited cell adhesion, migration and invasion in the hepatocellular carcinoma cells (Li et al., 2014). It could relieve atopic dermatitis-like skin symptoms by suppressing NF-κ B and STAT1 and inhibiting TNF-α/IFN-γ-induced TARC expression (Choi et al., 2014). In addition, platycodin D could inhibit the production of IL-6 and TNF- α to cure rheumatoid arthritis (Kwon et al., 2014). Platycodin D was also spermicidal, contraceptive (Lu et al., 2013), and anti-atherosclerotic (Wu et al., 2012). It has also been reported that platycodin D could be an alternative treatment of obesity by down-regulating lipid accumulation (Lee et al., 2012) and that platycodin D was identified as active component with anti-HCV activity (Kim et al., 2013). However, there was no report on the anti-*M. pneumoniae* property of Platycodin D.

This study was designed to investigate the anti-*M. pneumoniae* effects of Platycodin D and the related cell growth recovery after anti-*M. pneumoniae* treatment in the infected model organisms. *M. pneumoniae*–infected BALB/c mice and A549 type II pneumocytes from human-derived epithelial cell line were used to prove that Platycodin D could not only inhibit *M. pneumoniae in vitro* but also show the same effect *in vivo*. Our study presented a comprehensive view of Platycodin D, which would illuminate the main pharmacological action of Qinbai as a new clinical Chinese Medicine.

## Materials and methods

### Ethics statement

This study was carried out in strict accordance with the Guide for the Care and Use of Laboratory Animals of Heilongjiang Province. All research protocols were approved by the Animal Experiment Ethic Committee in Heilongjiang Academy of Chinese Medicine Sciences (Permit Number: [2011] 93). All surgeries were performed under sodium pentobarbital anesthesia, and all efforts were made to minimize suffering. Mice were humanely sacrificed via cervical dislocation.

### Materials

The PPLO Broth was purchased from BD, USA. Fresh yeasts were purchased from Angel Yeast Company, China. The RPMI 1640 media and Fetal Bovine Serum (FBS) were purchased from Hyclone, USA. Quantitative Diagnostic Kit for *M. pneumoniae* DNA was purchased from Da-An Gene, China. The Trizol was purchased from Ambion, USA. The 6-well plates were purchased from Corning, USA. Platycodin D was purchased from Chengdu Herbpurify Co. Ltd, China

### Platycodin D preparation

Platycodin D was purchased from Chengdu Herbpurify Co. Ltd (20 mg, J-013-120321, HPLC ≥ 98%). The 10 mg/ml Platycodin D solution was prepared with ultra-pure water and was stored at −20°C for late use. At the time of use, the stock solution was diluted with PPLO media supplemented with 20% fetal calf serum.

### *M. pneumoniae* and cell culture

*M. pneumoniae* (ATCC 15531) purchased from American Type Culture Collection was grown at 37°C in PPLO–yeast-extract–glucose-penicillin media supplemented with 20% fetal calf serum. 10^6^ ccu *M. pneumoniae* were used in the following experiments. A549 cells were cultured in 1640 medium with 10% fetal calf serum and 5% CO^2^ at 37°C, and were subcultured every 3–4 days by trypsinization.

### CCU

Color Change Unit (CCU): Tenfold dilution method was used to measure *M. pneumoniae* concentration in the original media. 1 ml medium containing 100 ul *M. pneumoniae* went through Tenfold dilutions with 10 bottles. The samples were incubated at 37°C for 1 week. One ccu is defined as the reciprocal of the first dilution that showed a stable color change.

### Cell infection

10^6^ ccu *M. pneumoniae* suspensions were used to infect 10^5^ A549 cells for 4 h before media supernatant was removed with unbound *M. pneumoniae*. Cells were divided into control (non-infected cells), model (infected cells without treatment) and treated groups (infected cells treated with 16 ug/ml or 32 ug/ml Platycodin D for 3d). *M. pneumoniae* DNA was quantified in each group.

### Animal infection

BALB/c mice (weighted 20 ± 1 g) were purchased from Laboratory Animals Center of Jilin University and were kept in GLP room at 25°C with 60% humidity. The mice were randomly divided into three groups, control group (non-infected mice), model group (infected mice treated with saline), and experimental group (infected mice treated with Platycodin D) (10 mice for each group). The model and experimental groups were inoculated with 20 ul 10^6^ ccu *M. pneumoniae* for 3d before the mice in experimental group were gavaged daily with 5 mg/kg.d (low dose) or 10 mg/kg.d (high dose) Platycodin D for 6d, while mice in control and model groups were given equal volume of saline. Same amount of lung homogenate were collected from each group for *M. pneumoniae* DNA quantification.

Preparation of High-dose Platycodin D on the mice (10 mg/kg.d): 5.1 g Platycodon grandiflorum/d (conversion of the Qinbai dose used in human treatment) × 1.5% (extraction percentage) × 0.0026 × 50.

Preparation of Low-dose PlatycodinD (5 mg/kg.d): 1/2 High dose Platycodin D.

### Minimal inhibition concentration (MIC)

Double dilution methods were used to determine the MIC of Platycodin D. If Platycodin D concentration reaches the MIC level, *M. pneumoniae* metabolism will be inhibited, resulting in no change of color in the sample. 1 ml medium containing 100 ul 10^6^ ccu *M. pneumoniae* was treated with 128 ug/ml Platycodin D initially and the mixtures went through double dilutions with Platycodin D concentration ranging from 128 to 2 ug/ml. After the color in the control media turned from red to yellow, the resulted mixtures were collected and were analyzed with real-time quantitative PCR (BIOER, Line Gene 9600, China).

### MTT

Cell proliferation was assessed by the MTT 3-(4,5-dimethylthiazol-2-yl)-2,5- diphenyltetrazolium bromide assay. Infected cells in 96 well plate (4 × 10^3^cells/well) were treated with 16 μg/ml or 32 μg/ml Platycodin D for 3 days, and non-infected cells were treated with 2–512 μg/ml Platycodin D for 3 days. The resulted cells were then incubated with 0.5% MTT reagent for 4 h at 37°C before they were treated with 150 ul of DMSO for 10 min (Twentyman and Luscombe, 1987). Absorbance at 490 nm was measured using a Tecan Infinite M200 PRO enzyme-labeled instrument.

### Real-time quantitative PCR

Quantitative Diagnostic Kit for *M. pneumoniae* includes a DNA extraction kit and an amplification kit (probe and primers). The measurement was carried out according to the manufacturer's protocol. Briefly, lung homogenate or cells of the 3 wells and DNA extract buffer were mixed in 100°C water for 10 min before the mixture was centrifuged at 12000 rpm for 5 min. The supernatant was used for the following PCR reaction. The PCR reaction conditions were as follows: initial denaturation at 93°C for 2 min, followed by 10 cycles of 93°C for 45 s and 55°C for 1 min, and another 30 cycles of 93°C for 30 s and 55°C for 45 s.

### Surviving cells count

The surviving cells were measured for control, model group and experimental groups (16 μg/ml and 32 μg/ml Platycodin D groups respectively). An equal volume of Trypan blue dyes was added to each well and cells were counted by cell count chamber under the light microscope. Cell concentration was calculated with the following formula: cell amount/ml = ∑ cell counted in 4 large squares/4 × dilution factor × 10^4^.

### Histopathology evaluation of the lung tissues and cells

A549 cells were fixed with 10% formalin for 48 h before they were stained with hematoxylin and eosin(HE). Fixed lung tissues were embedded in paraffin and then were cut into 5 μm slides. Tissue samples were stained with HE after they were hydrated with xylene and alcohol. Microscopic analyses were done using Olympus microscopy (BX41-DP72) at 20X magnification. The data were presented in a scoring system for histopathology inflammatory as described previously (Cimolai et al., 1992).

### Statistical analysis

All above experiments were carried out in duplicates. Data were presented as mean ± SD and were analyzed with paired *t*-tests. *P* < 0.05 was considered statistically significant.

## Results

### Cytotoxicity effect of Platycodin D

**Figure 1:**
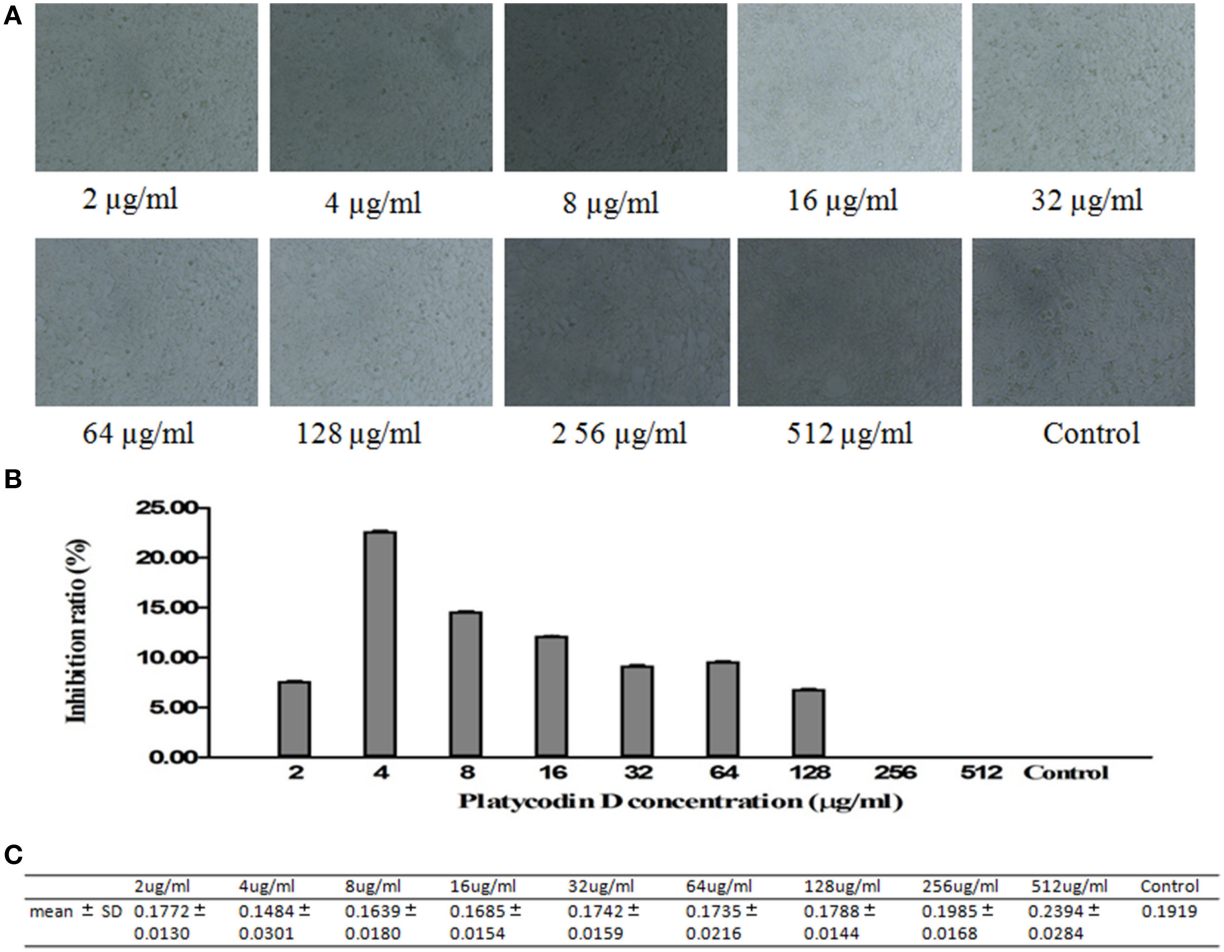
**Cytotoxicity effect of Platycodin D monitored by MTT assay**. The growth of A549 cells in 96-well plate was assessed according to the absorbance at 490 nm. **(A)** Cell images under microscopy. **(B)** MTT assay. **(C)** Statistical Analysis. Experiments were repeated twice. Data were presented as mean ± SD, *P* > 0.05.

MTT method was used to investigate the cytotoxicity effect of Platycodin D. Cells were divided into control and tested groups, with tested groups treated with 2–512 μg/ml Platycodin D for 3 days. Results showed that Platycodin D wasn't cytotoxic to cell growth with the concentration in the range of 2–512 μg/ml. Minor change on the cell inhibition ratio was observed with Platycodin D at 2–128 μg/ml, but this cell inhibition difference between control and tested groups was not statistically significant (*P* > 0.05) (Figure 1).

### The anti-*M. pneumoniae* property of Platycodin D

#### MIC

**Figure 2:**
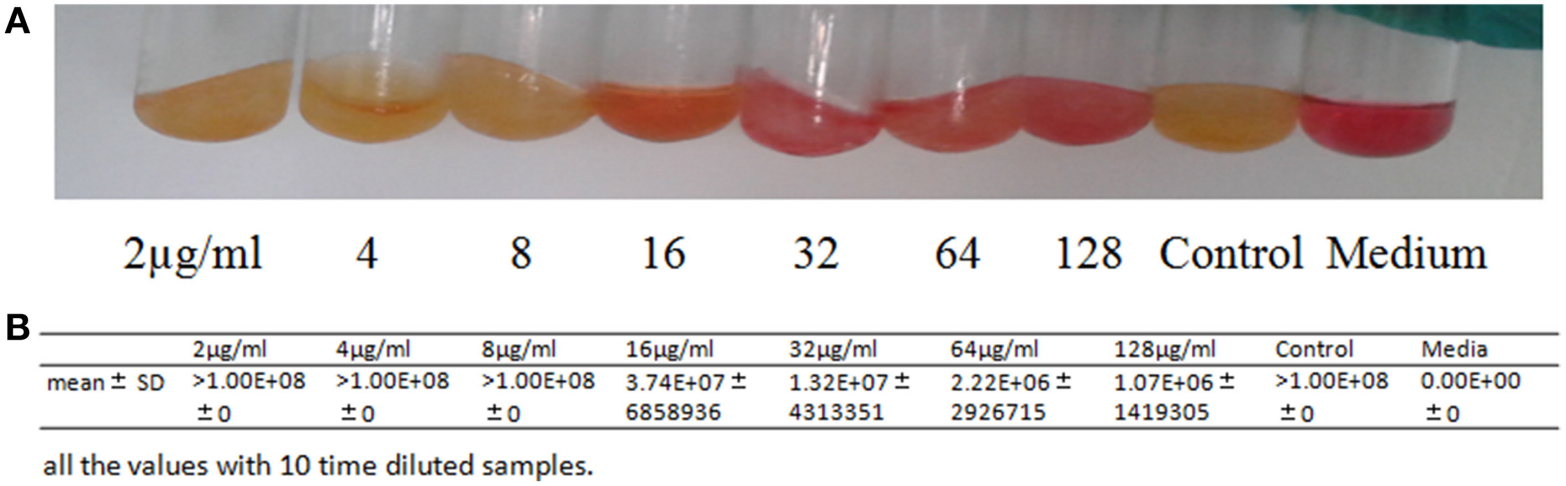
***In vitro* activity of Platycodin D against *M. pneumoniae*. (A)** The antimicrobial tests with the tube double dilution method. **(B)** Statistical Analysis of the the antimicrobial tests with Real-time quantitative PCR method.

*M. pneumoniae* were incubated at 37°C with Platycodin D ranging from 2 to 128 μg/ml (with serial double dilutions). Resulted mixtures were not collected until the red phenol turned into yellow in the control group. Gene expression was then measured with real-time quantitative PCR. Absolute standard curves of the Quantitative Diagnostic Kit for *M. pneumoniae* DNA are a linear range of 10^3^–10^8^ genome copies/ml. When bacteria concentration is more than 10^8^ genome copies/ml, quantification results in 10^10^ copies/ml. Platycodin D had a reasonably good inhibition capability against *M. pneumoniae*, with a MIC of 16 μg/ml (Figure 2). Our results suggested that Platycodin D would be a potential candidate for *M. pneumoniae* treatment.

#### Inhibition effects of Platycodin D on infected A549 cells

**Figure 3:**
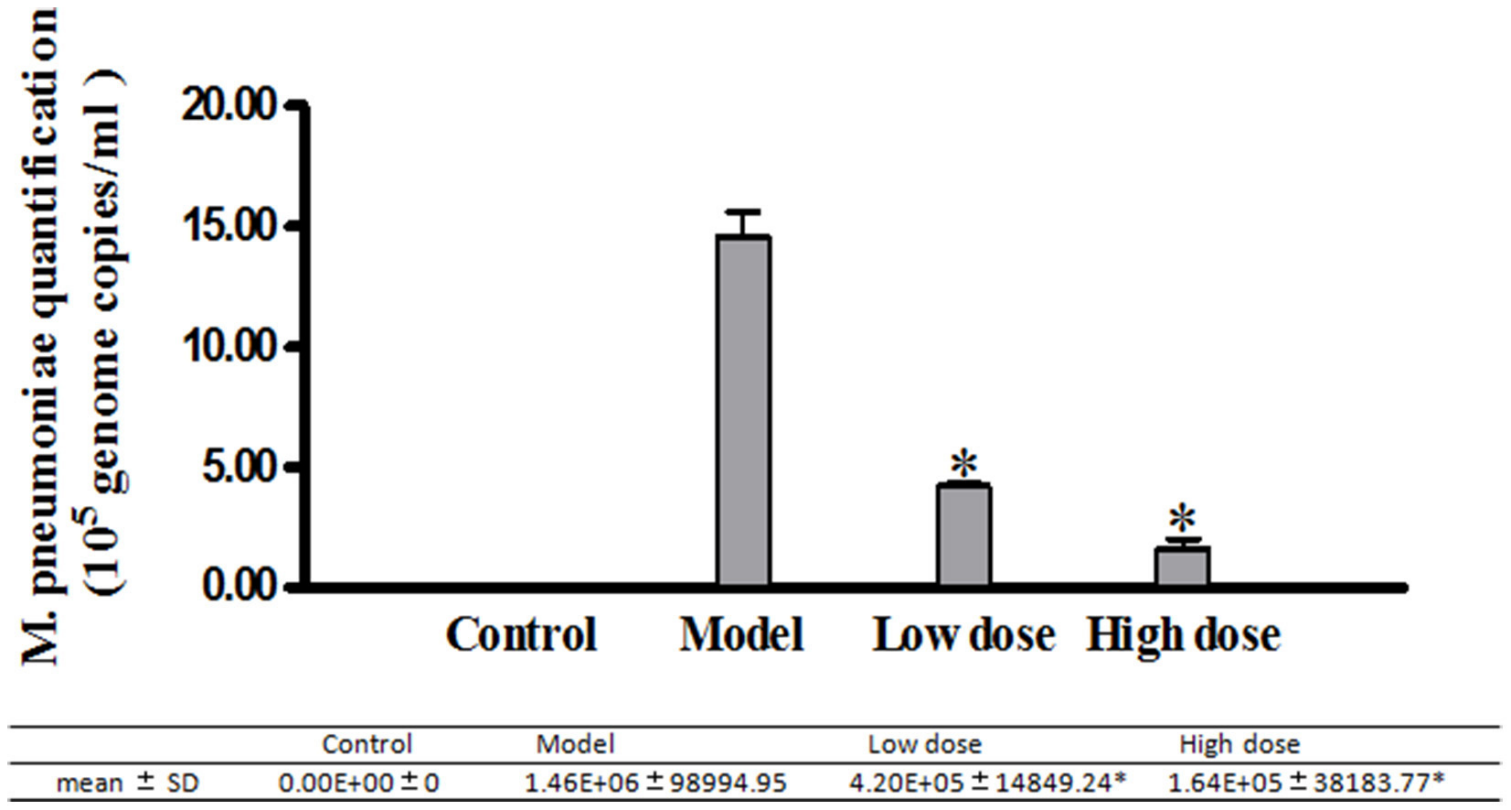
**Real-Time PCR analysis of *M. pneumoniae* amount in A549 cells**. Infected cells were treated with 16 μg/ml or 32 μg/ml of Platycodin D for 3 d. DNA levels were compared for those two groups. Experiments were repeated twice. Data were presented as mean ± SD, *p < 0.05.

A549 cells in different groups infected with *M. pneumoniae* and treated with Platycodin D were harvested and analyzed for the anti-*M. pneumoniae* effect of Platycodin D. Our results indicated that *M. pneumoniae* count less in the group treated with high dosage of Platycodin D than in the group treated with low dosage of Platycodin D compared to model (Figure 3).

#### Inhibition effect of Platycodin D in infected BALB/c mice

**Figure 4:**
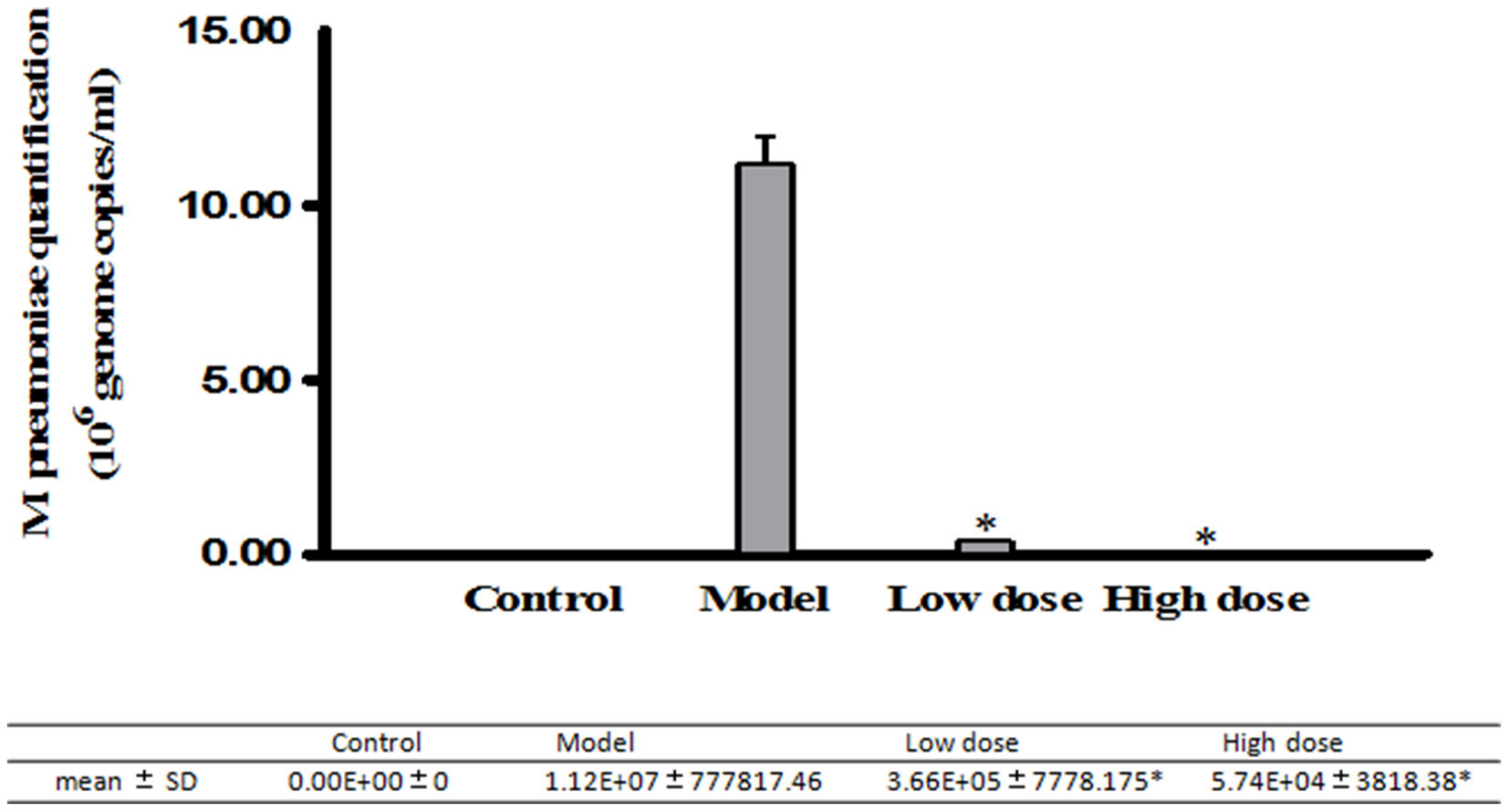
**Real-Time PCR analysis of *M. pneumoniae* amount in the mice**. Infected mice were gavaged once daily with 5 mg/kg/d or 10 mg/kg/d of Platycodin D for 6 d. DNA levels were compared between these three groups. Experiments were repeated twice. Data were presented as mean ± SD, ^*^p < 0.05.

Real-Time PCR was used to analyze DNA levels of *M. pneumoniae* in BALB/c mice treated with low or high dosage of Platycodin D. Our results indicated that the amount of *M. pneumoniae* dropped in both treated groups compared to the model with the high-dose-Platycodin-D -treated group dropping the most (Figure 4).

### Influence on the cell growth after anti-*M. pneumoniae* treatment

#### Surviving cells quantification in the infected A549 cell

**Figure 5:**
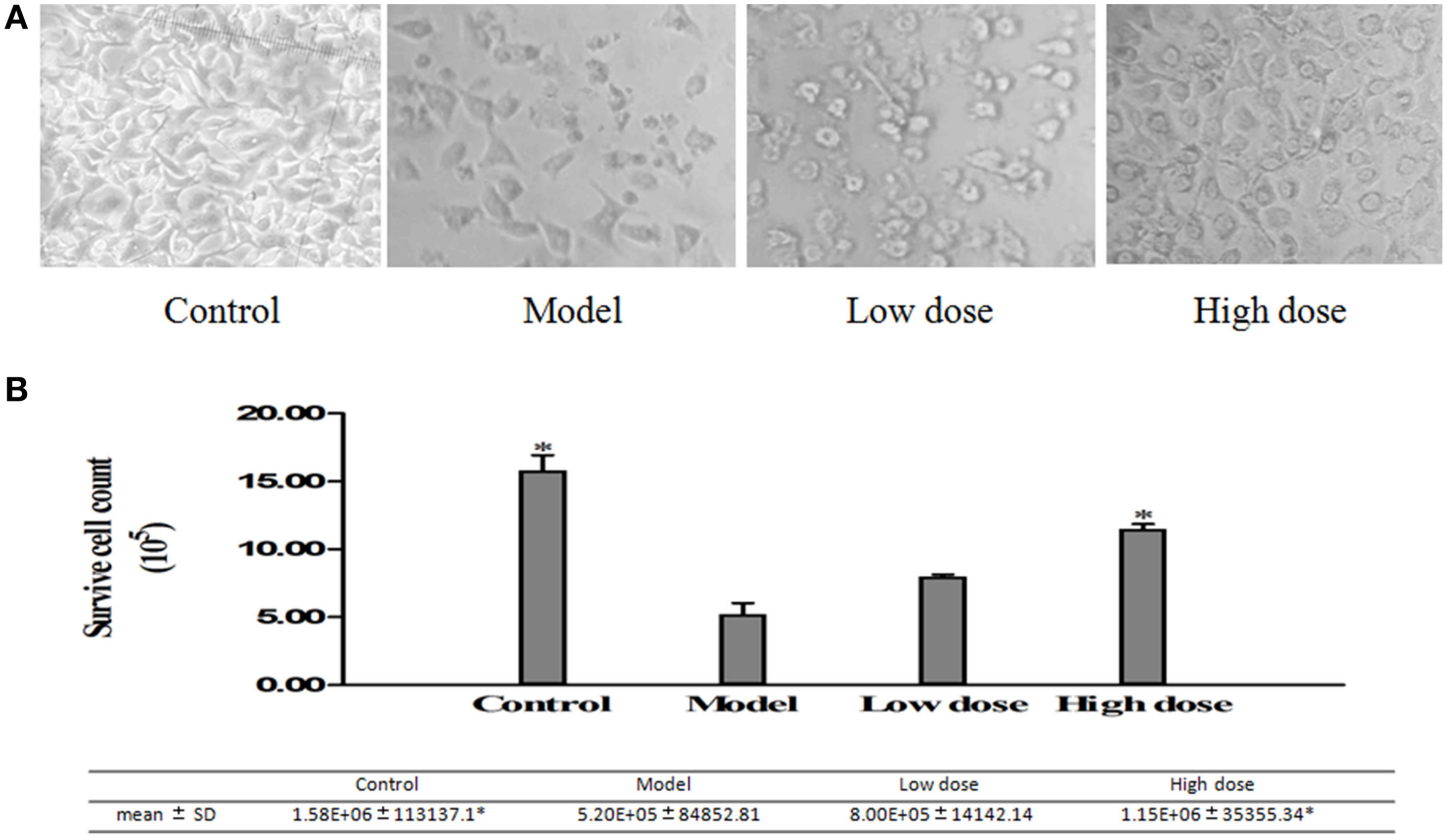
**Surviving cells quantified with images taken under the light microscope**. **(A)** Cell images under microscopy. **(B)** Surviving cells quantified by cell counting chamber. Experiments were repeated twice. Data were presented as mean ± SD, ^*^p < 0.05.

The cell amounts were measured with the Trypan Blue stain method, in addition to the cells imaging method under microscopy at 20X magnification. The cell count increased meaningfully in both high-dose (32 μg/ml) and low-dose (16 μg/ml) Platycodin D groups compared to the model group (Figure 5).

#### Viability of infected A549 cells treated with Platycodin D

**Figure 6:**
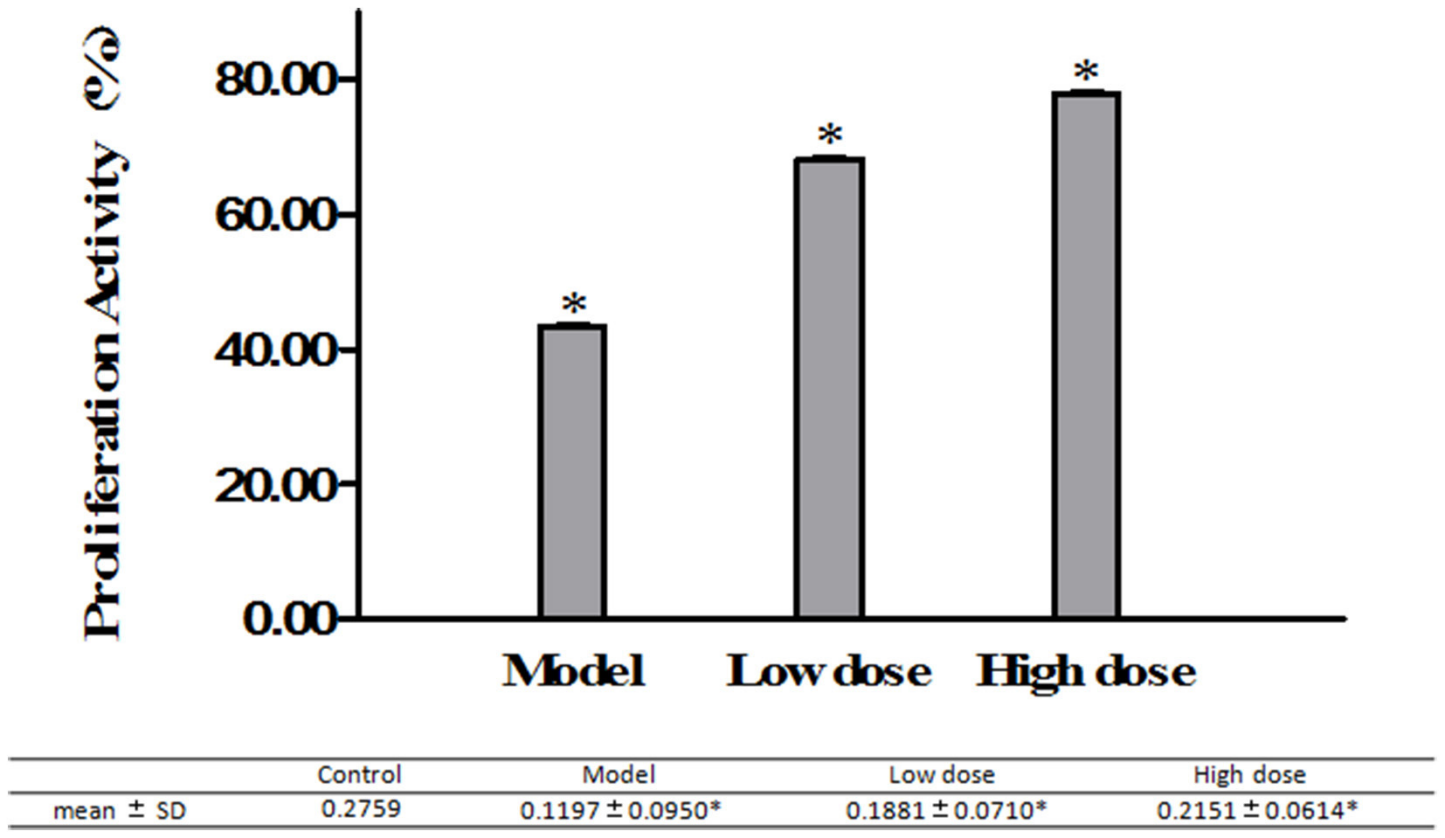
**A549 cell growth monitored by MTT assays**. Cells treated with 16 μg/ml or 32 μg/ml Platycodin D for 3d, compared with the control group. The growth of *M. pneumoniae*-infected A549 cells in 96-well plate was assessed based on the absorbance at 490 nm. Experiments were repeated twice. Data were presented as mean ± SD, ^*^P < 0.05.

MTT assay was used to investigate the effect of Platycodin D on the growth of *M. pneumoniae*-infected A549 cells. Cell proliferation results showed that treated groups had higher cell growth than the model group did, with the high dose group with the highest cell growth (Figure 6).

#### HE staining in A549 cells

**Figure 7:**
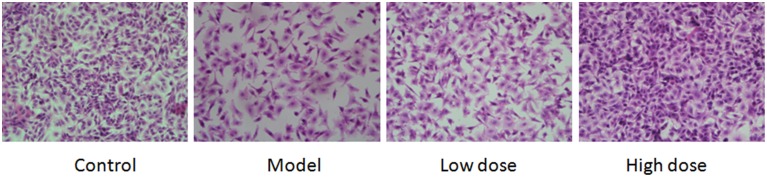
**Results of pathological changes in *M. pneumoniae*-infected A549 cells**. Cells treated with 16 μg/ml or 32 μg/ml Platycodin D for 3d, compared with the control group. Cells were imaged at 40X. Experiments were repeated twice.

Infected A549 cell HE staining results showed that cell density dropped while apoptosis increased in the model. In addition, cell hydropic degeneration, pale cytoplasm, intracytoplasmic vacuoles, and indistinct cytoplasmic borders were also observed in the model. There was a slightly decrease of the cell density and increase in apoptosis and cell hydropic degeneration in the low dose group while high dose group was similar to the control group (Figure 7).

#### HE staining in the lung tissue

**Figure 8:**
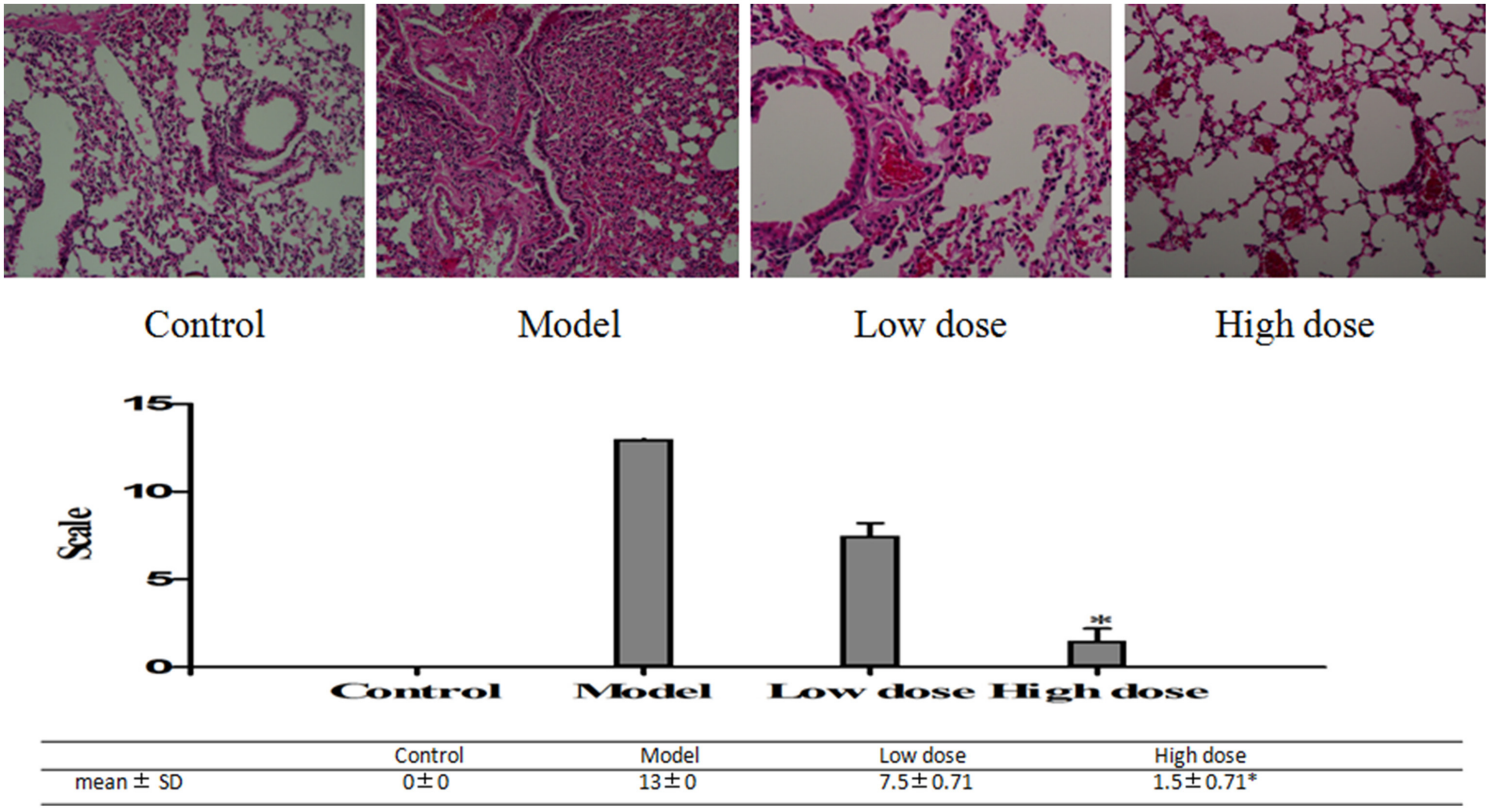
**Pathological changes of lung tissues in *M. pneumoniae*-infected BALB/C mice**. Histopathological changes of the slices on a scale from 0 (no change) to 26 (maximum inflammation) based on peribronchiolitis, perivasculitis, interstitial pneumonitis, and alveolitis. Mice treated with 5 mg/kg/d (low dose group) or 10 mg/kg/d (high dose group) Platycodin D for 6d, compared with the control. Slices were imaged at 20X. Experiments were repeated twice. Data were presented as mean ± SD, ^*^p < 0.05.

HE staining on lung fractions of these BALB/c mice was carried out to assess the changes in morphology. HE staining score was higher in the model group than in the treated groups. Desquamating of the epithelial cells, thickening of the peribronchovascular interstitium, collapse of alveolar walls, and congestion of capillaries with excess red blood cells were observed in the model group. In the low dose group, there were some desquamating of the epithelial cells, collapse of alveolar walls and the congestion of capillaries with excess red blood cells, but the situation was much lighter in the high dose group (Figure 8).

## Discussion

Platycodon grandiflorum, one of top 40 Chinese medicines in annual sales, has been widely used to treat various diseases in Traditional Chinese Medicine for thousands of years for its immunomodulatory, anti-inflammatory, expectorant, and antioxidant capabilities (Song and Qi, 2006). Its main active ingredients include triterpenoid saponin, flavanoid, phenolic compounds and fatty acids. Its monomers include Platycodin A, B, C, D, D2, D3, and Polygalacin D, D2 (Jin, 2007). According to some recent studies, Platycodin D showed bioactivity in anticancer (Chun et al., 2013), antirheumatoid arthritis and immunomodulatory (Kwon et al., 2014), and antiobesity (Lee et al., 2012). It was also reported that Platycodin D could regulate the production and secretion of airway mucin to prevent inflammatory diseases, which might explain the traditional use of Platycodon grandiflorum as expectorants in treating inflammatory pulmonary diseases (Ryu et al., 2014).

However, the anti-*M. pneumoniae* property of Platycodin D remained to be explained. Our study presented the first complete description of such activities of Platycodin D. Firstly, in this study, the cytotoxicity effect of the Platycodin D was measured by MTT to prove its safety and test showed that Platycodin D wasn't cytotoxic to cell growth up to 512 μg/ml. Minor change on the cell inhibition ratio was observed with Platycodin D at 2–128 μg/ml, but this cell inhibition difference between control and tested groups was not statistically significant. The results of the cell images under microscopy further showed that there wasn't obvious difference between the control and treated groups in terms of morphology and amount. Secondly, MIC of Platycodin D was measured for anti- *M. pneumoniae* capability, with the actual MIC of the Platycodin D determined as 16 μg/ml. Qinbai is a compound that includes 6 constituents and auxiliary material. Platycodon D concentration in the 100 μg/ml Qinbai is far lower than 16 ug/ml. Therefore, our study suggests that the combination of the different constituents of Qinbai causes inhibition of *M. pneumoniae* proliferation and promotion of cell growth after anti-*M. pneumoniae* treatment.

In addition, DNA levels of *M. pneumoniae* were measured in both infected cultured cells and animals following the treatment with a low or high dose of Platycodin D. Until now, methods used in monitoring *M. pneumoniae* concentration in cells or tissue included immunofluorescence and PCR DNA test methods. Among those methods, PCR was the most used in research laboratories (Ossewaarde et al., 1996; Tang et al., 2000). In our study, DNA of *M. pneumoniae* was measured with the Real-Time PCR. Our results showed that *M. pneumoniae* DNA level was higher in the model group than in the treated groups (low-dose and high-dose groups). *M. pneumoniae* amount dropped in the treated groups when compared to the model group *in vitro*. In infected animal model, same results were observed with mycoplasma higher in the model group than in the low dose and high dose groups. The above results both *in vitro* and *in vivo* further confirmed that Platycodin D had an inhibition effect on *M. pneumoniae* proliferation.

The second purpose of this study was to investigate the effects of Platycodin D on the growth of infected A549 cells and lung tissues after anti-*M. pneumoniae* treatment. MTT was used to measure the cell growth after anti-*M. pneumoniae* treatment and results showed that the treated group had a much better cell proliferation than the model group. HE staining is usually used to observe the cell or tissue damages. Damages of lung tissue such as desquamating of epithelial cells, collapse of alveolar walls and the congestion of capillaries, could be displayed clearly by this method. Changes of cell morphology including apoptosis, cell hydropic degeneration, and indistinct cytoplasmic borders could also be measured in this way. Morphological changes of cells and lungs for different groups were presented in Figures 5, 6. The results showed that the treated group had a much better cell or tissue proliferation than the model group. Trypan Blue stain and MTT analyses yielded the same results.

Our studies demonstrated that Platycodin D not only inhibited *M. pneumoniae* proliferation and promoted cell growth after anti-*M. pneumoniae* treatment *in vitro* but also showed the same effects *in vivo*. Our work provided a solid foundation for future work in exploring mechanisms of this Traditional Chinese Medicine.

### Conflict of interest statement

The authors declare that the research was conducted in the absence of any commercial or financial relationships that could be construed as a potential conflict of interest.
